# Analysis of the Genetic Basis of Disease in the Context of Worldwide Human Relationships and Migration

**DOI:** 10.1371/journal.pgen.1003447

**Published:** 2013-05-23

**Authors:** Erik Corona, Rong Chen, Martin Sikora, Alexander A. Morgan, Chirag J. Patel, Aditya Ramesh, Carlos D. Bustamante, Atul J. Butte

**Affiliations:** 1 Division of Systems Medicine, Department of Pediatrics, Stanford University School of Medicine, Stanford, California, United States of America; 2 Program in Biomedical Informatics, Stanford University School of Medicine, Stanford, California, United States of America; 3 Lucile Packard Children's Hospital, Palo Alto, California, United States of America; 4 Department of Genetics, Stanford University School of Medicine, Stanford, California, United States of America; Dartmouth College, United States of America

## Abstract

Genetic diversity across different human populations can enhance understanding of the genetic basis of disease. We calculated the genetic risk of 102 diseases in 1,043 unrelated individuals across 51 populations of the Human Genome Diversity Panel. We found that genetic risk for type 2 diabetes and pancreatic cancer decreased as humans migrated toward East Asia. In addition, biliary liver cirrhosis, alopecia areata, bladder cancer, inflammatory bowel disease, membranous nephropathy, systemic lupus erythematosus, systemic sclerosis, ulcerative colitis, and vitiligo have undergone genetic risk differentiation. This analysis represents a large-scale attempt to characterize genetic risk differentiation in the context of migration. We anticipate that our findings will enable detailed analysis pertaining to the driving forces behind genetic risk differentiation.

## Author Summary

The environment humans inhabit has changed many times in the last 100,000 years. Migration and dynamic local environments can lead to genetic adaptations favoring beneficial traits. Many genes responsible for these adaptations can alter disease susceptibility. Genes can also affect disease susceptibility by varying randomly across different populations. We have studied genetic variants that are known to modify disease susceptibility in the context of worldwide migration. We found that variants associated with 11 diseases have been affected to an extent that is not explained by random variation. We also found that the genetic risk of type 2 diabetes has steadily decreased along the worldwide human migration trajectory from Africa to America.

## Introduction

Analyzing the impact of human migration on genetic disease susceptibility is critical to the understanding of complex disease. Moving to new environments causes adaptation, which can also affect disease susceptibility. The availability of methods to measure genomic differences between worldwide populations has increased our understanding of human migration. For example, a worldwide human relationships phylogenetic tree was constructed after genotyping over 50 worldwide populations [1]. This process has enabled researchers to characterize worldwide genetic variation and has provided information regarding migrations that founded entire populations [2], [3]. At the same time, Genome-Wide Association Studies (GWAS) have increased discoveries of disease-associated genetic loci. These developments have paved the way for studies investigating the effects of migration on the genetic basis of disease [4]–[6].

The human genome has been subjected to many selective pressures in recent history. They include changes brought about by the domestication of crops and animals, and the rise of urbanization [7]. These changes may increase the frequency of mutations that are beneficial in the new environment. They may also lead to disruptions of biological processes. When mutations confer a net increase in fitness, they are expected to increase in frequency in affected populations [8], [9]. A mutation increasing disease risk can accompany a beneficial mutation through linkage disequilibrium (LD).

Studies have shown that unlinked single nucleotide polymorphisms (SNPs) associated with a single phenotype may be affected as a group if the phenotype undergoes differentiation [10], [11]. While a large set of loci may increase susceptibility to complex disease, individual loci generally make modest contributions, and their effect sizes indicate that they would not be expected to decrease reproductive success [12]. This situation allows differences in the genetic basis of disease to build naturally via genetic drift. However, deviations from genetic drift are expected when environmental changes occur due to migration [13]. Such changes provide an opportunity to learn about factors elevating disease risk in multiple populations.

Adaptation to new environments may have caused genetic risk differences across many human populations. Despite the recent explosion of knowledge regarding disease-associated loci and the genetic structure of different world populations [14]–[19], few studies have examined population-based differences in the genetic risk factors for disease. Additionally, they have included only a modest number of diseases, populations, or genetic samples. For example, Myles et al. [6] genotyped 25 disease-associated SNPs in ∼1,000 individuals from 53 populations in the HGDP-CEPH Human Genome Diversity Cell Line panel [15]. The study measured allele frequency differences in the SNPs and concluded that while risk allele differentiation was unusually high in some cases, overall, disease SNPs were not more differentiated between populations than random SNPs. However, 25 SNPs may not be sufficient to determine whether disease-associated SNPs as a whole have undergone risk allele differentiation in worldwide populations. These 25 SNPs are a small subset of variants that influence a small number of specific diseases. Other studies have examined interactions between migration, selection, and disease. One examined the impact of selection on hypertension variants during the migration out of Africa [20]. Others reported increased allele frequency differentiation of type 2 diabetes variants [21]–[23].

It is also plausible that allele differentiation may have occurred in unexamined combinations of diseases and populations. One study probed 2,186 disease-associated SNPs in the HapMap CEU cohort and concluded that they were enriched for low-frequency alleles [24]. This study used a large number of SNPs, but was conducted on a single population. A study incorporating a comprehensive catalog of SNPs and a large genetically diverse cohort would implicitly place genetic risk in the context of migration and reveal worldwide genetic risk variation with respect to many diseases.

In a previous study, we surveyed 8,377 SNPs representing 437 diseases in 11 subpopulations. We found differences in risk distribution and protective alleles in different diseases across many subpopulations [25]. The current study examines how the differences in these distributions could have arisen. We analyzed correlations between genetic risk and migration trajectories. Our goal was to identify populations with different genetic dispositions to disease, and to pinpoint migrations preceding these differences. Our analysis highlights the role evolution has played in changing disease susceptibility across populations.

Importantly, our approach controls for genetic substructure within and among diverse populations [26]. To detect differences in the genetic basis of disease across migratory events, we integrated a large curated database of geographically-annotated, disease-associated SNPs with human variation measurements in 51 populations from the Human Genome Diversity Panel (HGDP). We controlled for established genetic similarity in subpopulations in order to detect differences in genetic risk exceeding those expected under genetic drift.

## Results

### Type 2 Diabetes

**Figure 1:**
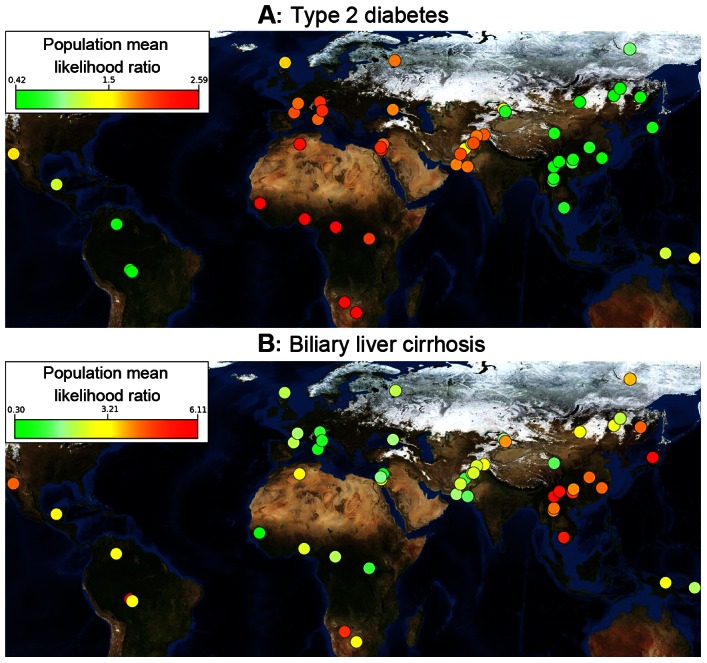
Differences in genetic risk among populations. Each population is ranked by risk, which is denoted by a color. Populations with the greatest risk are bright red, and those with the lowest risk are green. (A) Populations for East Asia and the Americas have lower genetic risk for type 2 diabetes than those from Africa and Europe. Genetic risk differentiation is sharply divided along major population migration events. Type 2 diabetes is represented by 16 SNPs. (B) Genetic risk for biliary liver cirrhosis is represented by 44 SNPs. Genetic risk peaks in East Asia and in the Karitiana population in South America. The background is a public domain world map from NASA Earth Observatory (http://eoimages.gsfc.nasa.gov/images/imagerecords/73000/73909/world.topo.bathy.200412.3×5400×2700.jpg); an interactive online tool is available at http://geneworld.stanford.edu using Google Maps technology.

Figure 1A summarizes our findings for type 2 diabetes on a world map. Consistent with our previous work [25], we found that African populations have the highest genetic risk for type 2 diabetes, followed by people from the Middle East, Europe, and Asia. The Mandinka population had the greatest risk, at a mean 0.95 log likelihood ratio (LLR), while the Surui population had the lowest risk (LLR: −0.87). This high-level view revealed stark differences in genetic risk between African and Asian populations. European and Middle-Eastern populations had an intermediate genetic risk for this disease.

These patterns identified worldwide trends of increases or decreases in genetic risk and show a high-level view of variation in genetic risk for different diseases. An interactive version of Figure 1A is available for over 100 diseases at geneworld.stanford.edu.

**Figure 2:**
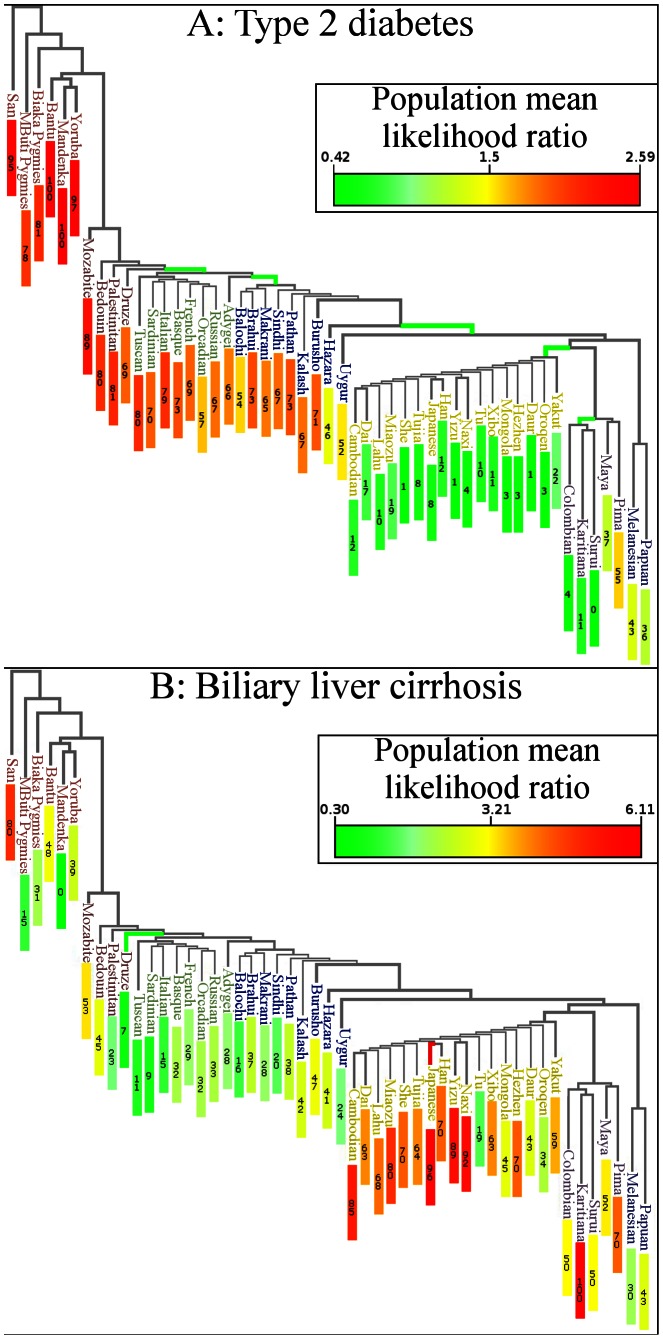
Genetic risk in the context of human relationships. The figure superimposes genetic risk for type 2 diabetes on a maximum likelihood phylogenetic tree based on the HGDP. Branch color indicates whether the subtree has shifted towards increased or decreased risk (q-value<0.05). Red∶increased risk; green∶decreased risk. Each colored branch also represents an *independent* genetic risk differentiation event as determined by a maximum likelihood model. Numbers in colored rectangles show percentile of genetic risk, with a zero being lowest. (A) Type 2 diabetes risk was decreased in East Asia and America. (B) Biliary Liver Cirrhosis shifted towards increased genetic risk in the Japanese population and towards decreased risk in the Druze population. The Karitiana population also showed borderline signs of genetic risk differentiation (q = 0.057).

Placing genetic risk on a map does not reveal close relationships between populations. Figure 2 is a worldwide phylogenetic tree displaying the relative genetic risk for each population in the context of population relationships. Relationships were inferred in a previous study [1] by analyzing 650,000 SNPs using a maximum-likelihood approach. Results from that analysis have been incorporated here. Each branch in the figure represents the ancestral population common to all populations below it.

Figure 1A and Figure 2A show that genetic risk for type 2 diabetes has undergone independent differentiation multiple times as humans migrated out of Africa to East Asia, and finally to the Americas. “Independent differentiation event” was defined as genetic risk differentiation occurring *de novo* in a population rather than via inheritance from an ancestral population. “Dependent risk differentiation” is a genetic risk inherited from an ancestor. Table 1 shows individual populations with genetic risk differences for type 2 diabetes that are larger than expected under genetic drift; however, it does not distinguish between dependent and independent genetic risk differentiation.

**Table 1:** List of populations with disease risk differentiation.

Disease	Population	Genetic Risk	World Genetic Risk	Q-value	SNPs
Alopecia areata	Mozabite	−3.546	−0.121	1.000×10^−5^	26
Biliary liver cirrhosis	Druze	−0.989	0.122	1.735×10^−2^	30
Biliary liver cirrhosis	Japanese	1.691	0.026	1.123×10^−2^	27
Bladder cancer	Tu	−0.535	−0.072	3.673×10^−2^	7
Inflammatory bowel disease	Balochi	−0.679	−1.051	1.000×10^−5^	11
Inflammatory bowel disease	Burusho	−0.886	−1.046	2.450×10^−2^	9
Inflammatory bowel disease	Makrani	−0.792	−1.048	1.533×10^−2^	10
Inflammatory bowel disease	Palestinian	−0.784	−1.056	3.433×10^−4^	11
Inflammatory bowel disease	Sindhi	−0.704	−1.051	3.433×10^−4^	10
Membranous nephropathy	French Basque	−1.876	−5.217	1.000×10^−5^	17
Pancreatic cancer	Biaka Pygmies	0.972	−0.215	2.602×10^−2^	9
Pancreatic cancer	Mbuti Pygmies	0.872	−0.194	2.780×10^−2^	9
Pancreatic cancer	Mandenka	0.877	−0.204	2.244×10^−2^	9
Pancreatic cancer	Yoruba	1.007	−0.207	1.429×10^−2^	9
Pancreatic cancer	Sardinian	0.244	−0.191	2.998×10^−2^	7
Pancreatic cancer	Cambodian	−0.644	−0.174	3.900×10^−2^	7
Pancreatic cancer	Japanese	−0.679	−0.165	2.702×10^−2^	7
Pancreatic cancer	Han	−0.601	−0.160	4.774×10^−2^	7
Pancreatic cancer	Naxi	−0.673	−0.175	4.871×10^−2^	7
Pancreatic cancer	Xibo	−0.722	−0.174	2.453×10^−2^	7
Pancreatic cancer	Daur	−0.732	−0.174	2.630×10^−2^	7
Pancreatic cancer	Maya	−0.783	−0.164	2.487×10^−2^	7
Systemic lupus erythematosus	French Basque	−2.76	−0.975	4.012×10^−2^	29
Systemic lupus erythematosus	Maya	1.354	−1.074	4.591×10^−2^	29
Systemic lupus erythematosus	Pima	1.882	−1.087	3.265×10^−2^	29
Systemic sclerosis	French Basque	−0.746	0.079	5.407×10^−2^	15
Systemic sclerosis	Maya	1.151	0.033	3.520×10^−2^	17
Type 2 diabetes	Bedouin	0.576	0.079	4.341×10^−2^	13
Type 2 diabetes	Cambodian	−0.643	0.11	2.577×10^−2^	13
Type 2 diabetes	Colombian	−0.787	0.114	3.659×10^−2^	12
Type 2 diabetes	Daur	−0.839	0.111	2.653×10^−2^	13
Type 2 diabetes	Han	−0.646	0.135	2.098×10^−2^	13
Type 2 diabetes	Hezhen	−0.806	0.11	1.918×10^−2^	13
Type 2 diabetes	Japanese	−0.723	0.126	1.734×10^−2^	13
Type 2 diabetes	Lahu	−0.674	0.11	4.826×10^−2^	13
Type 2 diabetes	Mongola	−0.807	0.111	3.877×10^−2^	13
Type 2 diabetes	Mozabite	0.749	0.083	2.183×10^−2^	15
Type 2 diabetes	Naxi	−0.78	0.11	2.051×10^−2^	13
Type 2 diabetes	Oroqen	−0.803	0.111	1.853×10^−2^	13
Type 2 diabetes	Palestinian	0.6	0.077	2.470×10^−2^	13
Type 2 diabetes	She	−0.834	0.111	1.874×10^−2^	13
Type 2 diabetes	Tu	−0.681	0.11	2.012×10^−2^	13
Type 2 diabetes	Tujia	−0.721	0.11	2.401×10^−2^	13
Type 2 diabetes	Xibo	−0.668	0.109	2.576×10^−2^	13
Type 2 diabetes	Yakut	−0.466	0.116	4.458×10^−2^	13
Type 2 diabetes	Yizu	−0.841	0.111	2.074×10^−2^	13
Ulcerative colitis	Balochi	−0.525	−1.778	1.000×10^−5^	27
Ulcerative colitis	Palestinian	−1.052	−1.784	9.183×10^−2^	29
Ulcerative colitis	Sindhi	−0.749	−1.772	1.025×10^−2^	27
Vitiligo	French Basque	0.339	−0.467	2.653×10^−2^	8
Vitiligo	Russian	0.239	−0.466	3.673×10^−2^	8

The populations in this table have undergone disease risk differentiation exceeding what would be expected by genetic drift for the diseases listed. Population-specific and overall genetic risks are shown. Genetic risk is the mean combined LLR for all SNPs associated with a disease. The q-value corrects for the number of populations tested. Certain diseases, such as bladder cancer, showed localized genetic risk differentiation while worldwide trends were evident for others, like pancreatic cancer and type 2 diabetes.

**Figure 3:**
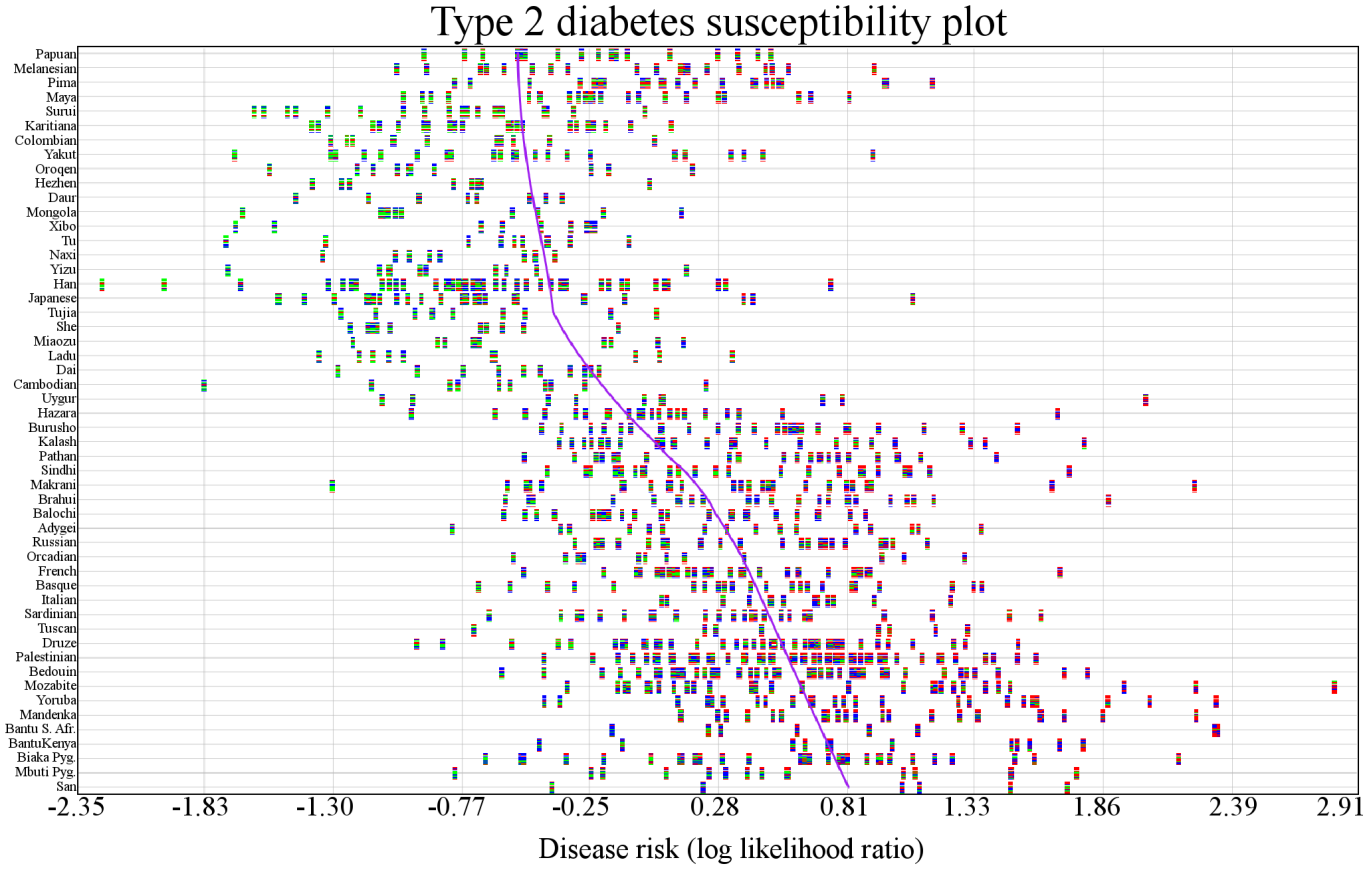
Variability in genetic risk for type 2 diabetes. Individuals are represented by vertical multicolored rectangles. Individual bars in each rectangle represent one of the 16 SNPs associated with type 2 diabetes. Bar colors indicate the following: red: homozygosity for a risk allele; blue∶heterozygosity; green∶homozygosity for a protective allele; white: missing genotype. The order of the SNPs is preserved across individuals. The x-axis shows genetic risk and the y-axis shows each population. The purple line is a locally weighted linear regression curve displaying the general direction of disease susceptibility as populations migrated from West to East. Genetic risk is lower in East Asians.

We used a maximum likelihood method to identify branches in the phylogenetic tree representing independent genetic risk differentiation events in Figure 2A. The log-likelihood of having only one event for type 2 diabetes was l_1 = _−108.848. The log-likelihoods for 2,3,4,5, and 6 events were l_2_ = −66.6083, l_3_ = −40.7165, l_4_ = −33.7324, l_5_ = −26.8371, l_6_ = −23.3415. We used the likelihood ratio test to determine the number of branches undergoing genetic risk differentiation independently that exceeded what would be expected under genetic drift. This test allows for the calculation of a p-value for ***n*** independent branches by converting two log-likelihood scores to a χ^2^ variable as follows: 2(l_n_−l_n−1_)∼χ^2^_1_. The p-value for 2 branches versus 1 branch undergoing independent genetic risk differentiation for type 2 diabetes was less than 1.00×10^−16^, meaning there was evidence for more than one independent genetic risk differentiation event. The p-values for 3, 4, 5, and 6 branches were 6.30×10^−13^, 1.86×10^−4^, 8.19×10^−3^, and 5.64×10^−2^, respectively. There was evidence for 5 distinct genetic risk differentiation events (highlighted in green in Figure 2A). Figure 3 shows the genetic risk of all 1043 individuals. Each type 2 diabetes-associated genotype in each individual is displayed. The x-axis shows individual genetic risk; the y-axis corresponds to West (bottom) to East (top) migration. The figure shows that genetic risk for type 2 diabetes decreases as populations move into East Asia. The figure shows that genetic risk decreases steadily, as opposed to being caused by a single genetic risk differentiation event.

Analysis of individuals showed that a person in the Mozabite population (HGDP1255) had the highest genetic risk (LLR:2.81), and an individual in the Han population (HGDP1291) had the lowest risk (LLR: −2.21).

The effect size of each individual variant is not necessarily the same in each population [27]. While the genetic risk of disease is currently computed using all available GWASs, there is a well-known European bias, as most GWASs are based on European-derived populations [28]. Table S1 displays the populations in which SNPs associated with type 2 diabetes in this study have been replicated. In order to ensure that effect sizes unique to European populations were not solely responsible for observed levels of genetic risk differentiation, the genetic risk for type 2 diabetes was recomputed using Asian-specific effect sizes for all variants. This was accomplished by using GWASs exclusively based on Asian populations. Table S2 compares the likelihood ratio computed using GWASs in which European populations are overrepresented with the Asian-specific likelihood ratio computed using Asian based GWASs. Table S3 compares the genetic risk estimates using all available GWASs and Asian-specific GWASs. Despite major differences in overall risk estimates, significant risk differentiation was still observed in Asian populations when risks were computed with Asian-specific GWASs.

### Biliary Liver Cirrhosis

Figure 1B shows the genetic risk of biliary liver cirrhosis (BLC) across worldwide populations. In contrast to type 2 diabetes, no worldwide trend is apparent. Figure 2B shows risk differences that were larger than expected under genetic drift (q<0.05). Branches were colored green if the deviation of a population below them shifted toward decreased risk compared to all other populations and red if the shift was toward increased risk. The maximum likelihood model was not applied to Figure 2B, as each population showing signs of genetic risk differentiation did not have descendant populations in the phylogenetic tree. The maximum likelihood test only distinguishes between inherited and independent genetic risk differentiation and is therefore only suitable for diseases displaying obvious worldwide deviation trends. Figure 2B shows that the Druze and Japanese populations have genetic risk differentiation exceeding what is expected under genetic drift. The Druze population shows significantly less risk, with only 28 out of 100,000 random draws showing less risk (q<0.05) than all other populations combined. The genetic risk in the Japanese population was higher, with only 18 out of 100,000 random draws showing a higher risk. Genetic risk differentiation in BLC was localized in one European and an Asian population.

**Figure 4:**
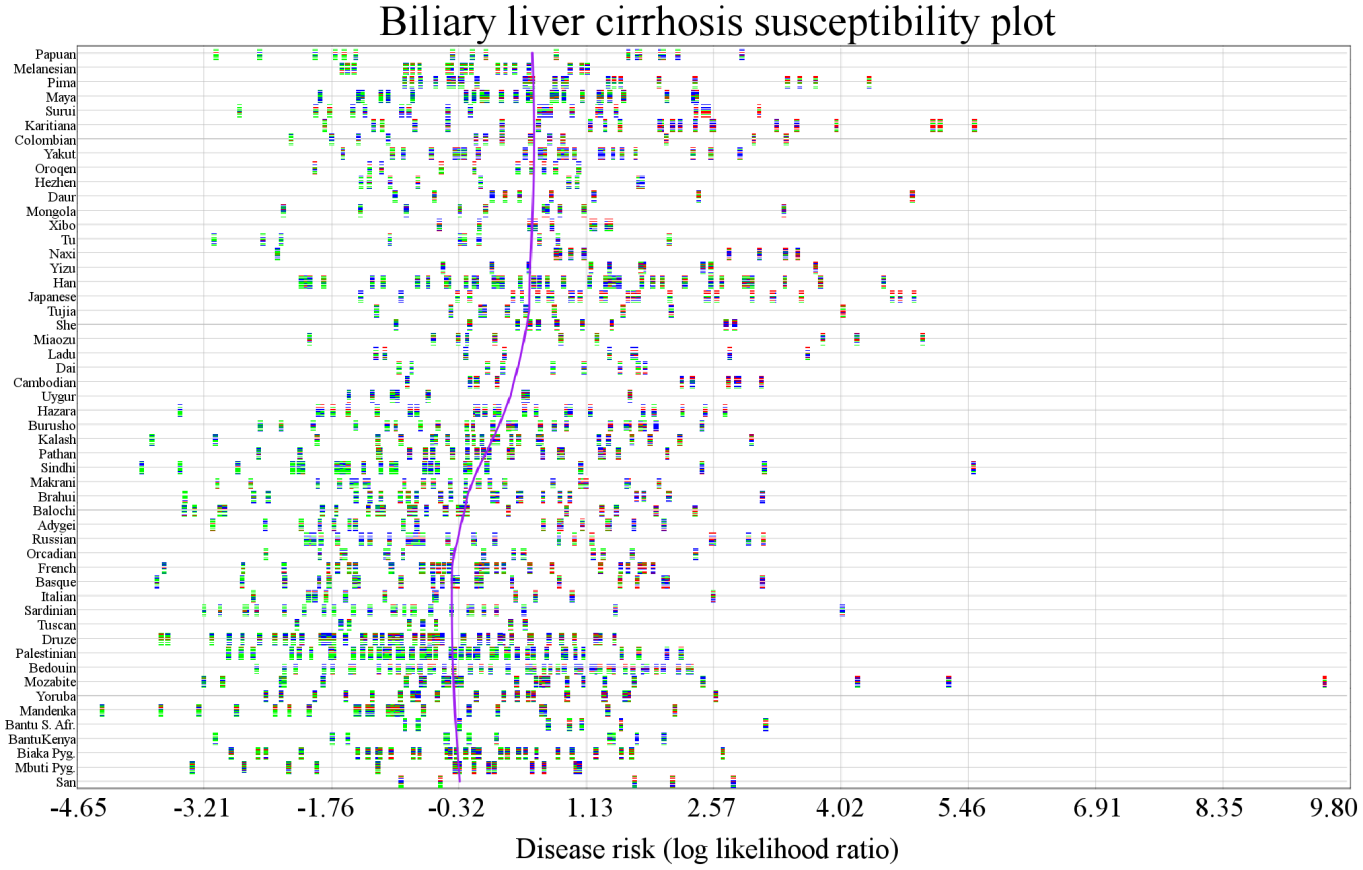
Variability in genetic risk in biliary liver cirrhosis. Individuals are represented by vertical multicolored rectangles. Bar colors indicate the following: red: homozygosity for a risk allele; blue: heterozygosity; green: homozygosity for a protective allele; white: missing genotype. The x-axis shows risk and the y-axis shows each population. The purple line is a locally weighted linear regression curve displaying the general direction of disease susceptibility as populations migrated from West to East. No trend is obvious, but risk appears to be higher in Cambodian, Yizu, Japanese, and San populations.

We examined genetic risk in individuals to expose potential outliers (Figure 4) and to visualize variation in genetic risk estimates across all individuals. The person with the lowest risk (HGDP1201) was from the Mandinka population and had a risk score (combined LLR) of −4.372. The person with the highest risk (HGDP1279) was from the Mozabite population and had combined LLR of 9.521. This person appears to be an outlier. The individual with the second highest genetic risk (HGDP998) was from the Karitiana population (combined LLR: 5.54).

### Other Diseases

**Figure 5:**
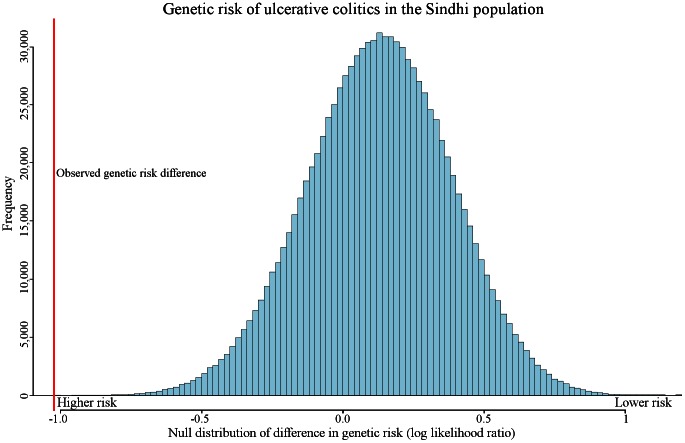
Expected amount of genetic risk differentiation in ulcerative colitis. Random variation caused by genetic drift may alter genetic risk between populations. The figure represents the expected amount of genetic risk difference between Sindhi and all other populations. A randomly generated genetic risk score was computed by randomly picking SNPs to represent ulcerative colitis (see Materials and Methods). The red vertical line represents the observed genetic difference between the Sindhi and all other populations combined. Only 15 out of 100,000 randomly generated genetic risk values had a larger genetic risk difference than the observed.

We observed genetic risk trends associated with migration in other diseases, including prostate cancer, alopecia areata, melanoma, asthma, neuroblastoma, polycystic ovary syndrome, and pancreatic cancer. One of the most extreme examples of genetic risk differentiation was observed in ulcerative colitis. Figure 5 displays the distribution of the expected amount of genetic risk difference for this disease between the Sinhdi population and all others. The red vertical line represents the actual observed genetic risk difference between the two populations. Only 15 out of 100,000 randomly generated genetic risk values had a larger ulcerative colitis genetic risk difference.

We found genetic risk differentiation in multiple worldwide populations for pancreatic cancer and other diseases (Table 1). Detailed information relating to genetic risk differences for many other diseases are available on (geneworld.stanford.edu).

Individual SNPs may have a large impact on overall levels of genetic risk differentiation (Text S1 and Figure S1). One notable case is rs13151961, associated with inflammatory bowel disease. Figure S1 shows examples of diseases in which a few SNPs have a disproportionate impact as well as examples in which all SNPs have a uniform impact on genetic risk differentiation.

### Fst and Differentiation

Our method can detect genetic-risk differentiation that is not captured by Fst calculations. Figure S2 shows that rank normalized Fst values, after binning with SNPs having matching allele frequencies, failed to detect population structure differentiation among SNPs associated with biliary liver cirrhosis. After combining p-values for each individual SNP, the combined p-value was 0.91. Our method detects genetic-risk differentiation in the Japanese and Druze populations (Figure 2B). Figure S3 shows the rank normalized Fst values for SNPs associated with type 2 diabetes. The combined p-value for the observed type 2 diabetes Fst scores was not significant (p = 0.30). Our method detects genetic-risk differentiation for multiple populations in type 2 diabetes, as can be observed in Figure 2A. Text S1 has additional details on the Fst analysis.

### Individual Outliers

Individuals may be outliers with respect to genetic risk, leading to false detection of genetic risk differentiation in a population. We defined outliers as individuals deviating >1.5 times the interquartile genetic risk range from the median genetic risk and removed them from each population-disease sets showing genetic risk differentiation (Table S4). Each disease showed strong signs of genetic risk differentiation after removing outlying individuals.

### Controlling for Association Ascertainment Bias

Disease-associated SNPs are likely to be biased towards genomic regions with higher LD levels where they are tagged more efficiently. We addressed this bias by resampling from SNPs reported to be associated with any phenotype (disease or not) as opposed to resampling from all SNPs. We discarded 614,563 and retained 46,192 of the genotyped SNPs in the HGDP cohort. Genetic risk differentiation was still detected after resampling exclusively from SNPs associated with a phenotype (Table S5).

## Discussion

Our findings place the genetic basis of disease susceptibility in the context of human migration and increase understanding of the role of population differentiation in complex disease. The HGDP-CEPH cohort represents 51 populations from around the world, with >650,000 genotyped SNPs per person. We combined this cohort with SNPs from our disease association database and were able to assess the genetic risk of disease of all populations in the HGDP-CEPH cohort. We demonstrated that differences in genetic risk for multiple diseases go well beyond what is expected by genetic drift. In addition, using a human population phylogenetic tree allowed us to incorporate a substructure of worldwide relationships.

A certain amount of variation can be expected due to random frequency variation in disease-associated genotypes. Genetic risk differentiation not caused by genetic drift is more likely to be caused by environmental adaptation, and provides insight into factors affecting disease susceptibility. Our method controls for random genetic risk variation by randomly sampling SNPs across the genome. When testing for significance in our model, the baseline was the expected amount of risk differentiation caused by genetic drift between a population of interest and all others. The low q-values in Table 1 are of particular interest, as they indicate that genetic drift is unlikely to account for the amount of observed genetic risk differentiation.

Genetic risk differentiation occurred in multiple populations in type 2 diabetes. In contrast, genetic risk differentiation in biliary liver cirrhosis was found in only two populations. Ulcerative colitis exhibits the single most extreme example of genetic risk differentiation (Figure 5). These three diseases capture the observable distinct genetic risk differentiation patterns.

### Admixture

While the HGDP is an important sampling of the worldwide distribution of genotypes, it is possible that some populations are admixed. Detecting genetic risk differentiation in admixed populations may be more difficult, as the levels of genetic risk would most likely be less extreme. The genetic risk for any two populations in which admixed individuals were erroneously sampled is expected to be somewhere between the population with the least and most genetic risk. This explains why controlling for admixture is unlikely to decrease the significance of our findings.

### Type 2 Diabetes

Multiple independent genetic risk differentiation events have occurred in various worldwide populations. Table 1 shows individual populations reported to have undergone or inherited an increase/decrease in genetic risk. We applied a maximum likelihood method to identify branches in a phylogenetic tree representing independent genetic risk differentiation events for type 2 diabetes (Figure 2A). Individuals migrate toward decreased genetic risk of type 2 diabetes as populations migrated East. Figure 3 shows that while there was great variability in risk within populations, there was still a steady decrease in susceptibility to type 2 diabetes.

### Biliary Liver Cirrhosis

There were 44 SNPs associated with biliary liver cirrhosis used in this study. We found genetic risk differentiation in the Japanese and Druze populations (Figure 2B). Risk was increased in the Japanese population. The genetic risk score (combined LLR) was 1.691, compared to 0.026 for all other populations combined. The q-value for such a large risk difference was 0.0112 (Table 1). Consistent with results in this study, our previous work shows significantly higher biliary liver cirrhosis risk in the Japanese population (p: 0.013) in the Hap Map III cohort [29].

### Ulcerative Colitis

Like all the diseases discussed in this study, ulcerative colitis has genetic and environmental components [30]. Our results suggest that genetic risk differentiation for this condition is increased in South Asian Sindhis (Figure 5). Previous studies have reported that prevalence rates are not necessarily correlated to significant increases in genetic risk [31], [32]. However, our results imply that some event affected pathophysiology of this disease in a particular population. The environment, as well as currently unidentified loci, may also affect the absolute risk. In addition to the Sindhi population, genetic risk differentiation was detected in the Palestinian and Balochi populations (Table 1).

### Cause(s) of Type 2 Diabetes Genetic Risk Differentiation

Specific environmental differences inducing genetic risk differentiation in type 2 diabetes and other diseases have not been found. However, there is evidence that climate, diet, and living conditions have led to them [33]. For example, exposure to viruses may have increased risk for type 1 diabetes [34]. Autoimmune diseases show disproportionate positive selection in a trajectory toward increased versus decreased risk [35]. This finding has given rise to speculation that viral epidemics are likely to have increased the risk of these diseases by selecting for an overactive immune system. It is also established that modern cultural changes can cause drastic differences in disease prevalence in related populations [36]. However, little is known about how these changes modify risk profiles and disease prevalence over time. This study provides critical clues for the foundation for future analyses.

### Detection of Disease Inter-Relationships

The concepts discussed here could be used to link diseases that may share pathophysiology and environmental triggers. It is possible that modulation of environmental features in different global regions changed the genetic risk of certain diseases. If an environmental feature affects multiple diseases, risk estimates for the diseases should correlate across the same populations, even if no common genetic basis is apparent. If the environment increases risk for one disease and decreases it for another, a negative correlation is expected. Finding diseases with genetic risk estimates correlated across worldwide populations would represent a novel and potentially highly informative approach to uncover shared pathophysiologies. This type of analysis would benefit from the largest possible catalog of genetic variants. For example, in the HGDP, East Asia is biased to detect more genetic risk differentiation than the Americas, due to the increased sensitivity that comes from 17 East Asian versus 5 American populations. In addition, full genome sequence analysis enables the inclusion of copy number polymorphisms and rare variants that may be found to contribute to complex disease susceptibility. As sequencing costs decrease, analyses expanding the scope of this study will occur.

## Materials and Methods

### Data and Cohorts

We investigated >650,000 SNPs from the HGDP-CEPH, which has DNA from 1043 individuals in 51 populations on 8 continents [15]. We also used the HapMap Phase 3 cohort to analyze 1.6 million SNPs from 11 populations [29]. Finally, we used VARIMED, a database of disease-associated SNPs [37]. VARIMED was built by curating 5,478 published studies with 4,573 disease associations. At the time of this study, it contained 67,678 unique phenotype-associated SNPs, of which 51,404 were associated with a disease. Of these, after filtering by p-value and other methods, 723 unique SNPs were on the Illumina genotyping array we used to represent disease phenotypes in the HGDP [15].

For this study, we used only GWAS SNPs that had been detected across ≥2 populations with p-values<10^−6^. SNPs were excluded if information about them was insufficient to compute likelihood ratios for the genotypes of associated SNPs. VARIMED was used to compute genetic risk estimates of the resulting diseases across all HGDP populations.

The HapMap Phase 3 cohort includes 11 populations with 1.6 million SNPs genotyped per person [29]. We used this cohort, in combination with results from previous work [25], to check our results in individual populations with elevated levels of genetic risk differentiation. All the methods with the exception of multiple hypothesis testing are applicable to this cohort.

### The Combined Likelihood Ratio As a Measure of Genetic Risk

In this paper, the *sample* genetic risk in a population is referred to as *genetic risk*. Any computation of a population's genetic risk is inaccurate, due to the inability to genotype all individuals in a population and the existence of many undiscovered disease-associated variants.

The likelihood ratio (LR) represents the effect size of a particular genotype on genetic disease risk. SNPs in linkage disequilibrium (R^2^≥0.2) in a population were excluded.

### Computing Genetic Risk of a Disease in an Individual

For a given bi-allelic SNP, there are three possible genotypes: homozygous for the major allele, homozygous for the minor allele, or heterozygous. The function **L(** ***g*** **)** maps the genotype ***g*** to the estimated likelihood ratio. The LR used in our calculations represents the weighted mean LR reported across all studies [37]. Each LR was weighted by the square root of the sample size in each association study.

The following is our method for combining multiple LRs from multiple GWASs. ***A*** is the vector of all sample sizes in each study. P(***g*** | disease is present)_i_ is the estimated probability that a genotype ***g*** is in the disease population of the ***i*** *^th^* GWAS. P(***g*** | disease is not present)_i_ is the estimated probability that a genotype ***g*** is present in the non-disease population of the ***i*** *^th^* GWAS.
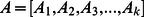


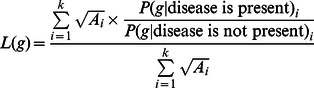



Once computed, the combined LR was used to compute the genetic disease risk for each individual, as follows. ***G*** is the vector of all genotypes in disease-associated SNPs in person ***m***.
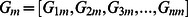



The predicted genetic risk ***r*** for person ***m*** is the log of the combined likelihood ratios for all disease-associated variants present in that person.
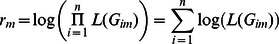



### Computing the Genetic Risk of Disease in a Population

The genetic risk for a population was the mean risk of all its members. Derived populations created by combining multiple populations in the HGDP were treated as single populations, and the following computations applied equally. ***S*** is the vector of a population.
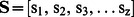



The predicted genetic risk ***R*** for an entire population ***P*** is the mean predicted risk for everyone in the population:
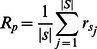



### Computing the Difference in Genetic Risk across Two Populations

In order to compare genetic risk (*D*) between two populations *A* and *B*, we subtracted the genetic risk of one population from the other's:
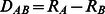



Population *A*'s risk for a disease is higher than *B*'s if *D_AB_* is positive and lower if *D_AB_* is negative.

### Modeling the Expected Difference in Genetic Risk

We constructed a distribution of the expected difference of genetic risk between two populations in order to see if the observed difference was larger than expected by random chance:.

The vector *H* represents all SNPs associated with a particular disease.
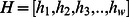



Our goal was to assess the significance of a difference in risk *D_AB_* across two populations *A* and *B*. Each element in *H* was replaced by a SNP randomly drawn from the entire set of SNPs in the two populations. The global major allele frequency of the randomly drawn SNP was drawn to match original SNP's global major allele frequency. In every case, the risk allele's major or minor allele status in the randomly drawn SNP matched that of the SNP it replaced. In addition, each SNP was placed in one of eight functional categories (frameshift, nonsense, missense, untranslated, near-gene, intron, coding-synonymous, or unknown). Each randomly drawn SNP also matched the functional category of the SNP it replaced in vector *H*.

Once all elements of *H* had been replaced, the genetic risk of all populations was recomputed, effectively assigning a randomly generated genetic risk score to each population. Since each population was assigned a genetic risk score from the same randomly drawn set of SNPs, the expected amount of correlation between genetic risk values among all populations was preserved. We created phylogenetic trees of our results with each branch representing a migration event. We computed the genetic risk difference of each migration event by subtracting the genetic risk of all descendant populations from the risk of all ancestral populations (those above the branch). Branches on the human phylogenetic tree created from the HGDP populations were tested for genetic risk differences. We computed the difference in risk between all ancestral and descendant populations. A phylogenetic tree of all the HGDP populations was used as described previously [1]. Each branch in the tree partitions an ancestral and descendant population. The ancestral population is made up of populations above a branch; the descendant population is below it. The expected difference in genetic risk between all possible ancestral and descendant comparisons was computed by randomly replacing all disease-associated SNPs by performing a random draw of *H* 100,000 times.

We computed a matrix representing 100,000 randomly generated phylogenetic trees and compared it with the observed phylogenetic tree in the context of genetic risk. Let *r_i,k_* represent the genetic risk difference between ancestral and descendant populations in branch *i* computed from the *k^th^* randomly generated *H*.
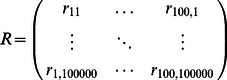



This matrix of 100,000 randomly generated phylogenetic trees was then compared to the observed tree. *O_i_* is the observed genetic risk difference between ancestral and descendant populations in the *i^th^* branch of the phylogenetic tree.
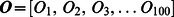



The probability of observing a genetic risk difference between ancestral and descendant populations in the *i^th^* branch is as follows:
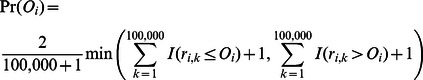



This equation gave a probability for a specific genetic risk difference observed on branch *i*. However, we tested multiple strongly correlated populations simultaneously. In addition, the genetic risk differences of nearby branches in the phylogenetic tree were strongly correlated due to genetic similarity between populations. One row in matrix ***R*** represented the genetic risk difference of all branches in the tree between ancestral and descendant populations in a single random draw. The genetic risk difference was calculated with the same set of SNPs in every branch in the phylogenetic tree in each row. This preserved the correlation of genetic risk among closely related populations and enabled us to detect independent genetic risk deviations.

To assess the significance of a p-value *α* in the context of the entire tree, we first computed the number of branches having equal or greater significance for genetic risk differentiation.

Significant genetic risk differentiation events were calculated follows. *N(α)* is the observed number of branches with a p-value for genetic risk differentiation at or below *α*.
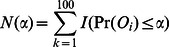



We next computed the expected number of branches with genetic risk differentiation at significance *α* from the matrix representing 100,000 randomly generated phylogenetic trees.
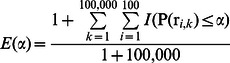

*Q(*α*)* (the q-value) for a branch with p-value *α* for genetic risk differentiation was:
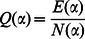



While *Q(* 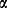 *)* can inform on the significance for a particular p-value, it does not estimate the total number of distinct genetic risk differentiation events in a phylogenetic tree. We estimated the most likely number of migration events independently contributing to observed genetic risk deviation in a phylogenetic tree using a maximum likelihood approach.

*P* is the vector containing all probabilities of the observed genetic risk difference between ancestral and descendant populations of each branch of tree *t*.
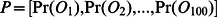



We defined a function that returns true if branch ***k*** is not a descendant of branch ***z***:
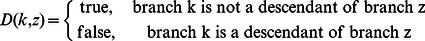



Branch *z* represents the branch in which an independent genetic risk differentiation event occurred. Applying the principle of maximum likelihood, the branch *z* that maximizes the likelihood function in tree *t* is shown below. The number of events refers to the number of independent genetic risk differentiation events.
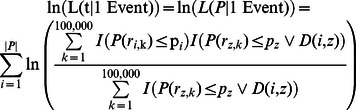


The log likelihood function produces the branch most likely to have undergone genetic risk differentiation, given that a single genetic risk differentiation event occurred. The ‘
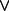
’ symbol represents a logical disjunction that returns *true* if one or more of the two operands is true. The branch that maximized likelihood was the one most likely to have caused the risk differentiation. However, there may have been multiple genetic risk differentiation events. This maximum likelihood method can be generalized to detect an arbitrary number of branches that underwent genetic risk differentiation.

Find the indices *z_1_, z_2_, …,z_y_* for branches that maximize the following likelihood function:
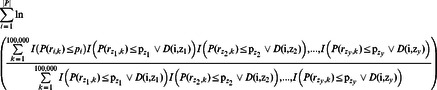



Due to the constraints this equation imposed, a very large number of random draws was required to produce an accurate estimate. All indicator functions in the numerator must return a non-zero value for the count to be incremented by one; this was computationally infeasible. In order to make the computation tractable, we made a simplifying assumption that only a single ancestral branch which has undergone *de novo* genetic risk differentiation (as opposed to an arbitrary combination of branches that have undergone *de novo* genetic risk differentiation) can pass a significantly modified genetic risk for a disease to a descendant branch. This led us to the method used for this study. The following calculation was used to find all branches with sufficient evidence of having undergone independent genetic risk differentiation.

Find the indices *z_1_,z_2_,…,z_y_* that maximize the following likelihood function:
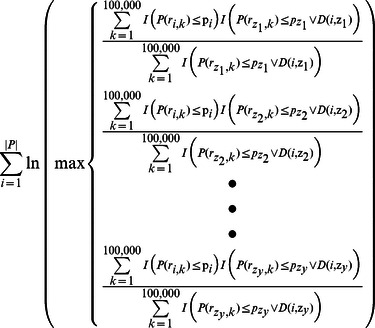



## Supporting Information

Figure S1 Impact of individual SNPs on genetic risk differentiation. The relative deviation from expected genetic risk attributable to each SNP is shown in each pie chart. The branch in the human phylogeny tree with the most significance for genetic risk differentiation was used to assess the impact of individual SNPs for each disease. The branch selected for alopecia areata includes European, Central South Asian, East Asian, Oceaniac, American, Palestinian, and Druze populations. The branch selected for inflammatory bowel disease includes the Brahui and Makrani populations. The branch selected for pancreatic cancer includes Central South Asian, East Asian, Oceaniac, and American populations. The branch selected for systemic lupus erythematosus includes the Mayan and the Pima populations. The branch selected for type 2 diabetes includes East Asian, Oceaniac, and American populations. The branch selected for ulcerative colitis includes the Brahui and Makrani populations. The branches selected for biliary liver cirrhosis, bladder cancer, and membranous nephropathy are the Druze, Tu, and French Basque populations, respectively. Each branch is compared to all other worldwide populations. Some SNPs have a disproportionate impact on genetic risk. An example is rs13151961, associated with inflammatory bowel disease.
(TIF)

Figure S2 Fst analysis for biliary liver cirrhosis. Global Fst values using the HGDP cohort were calculated for the 44 high confidence biliary liver cirrhosis SNPs used in this study. The Fst score for each SNP was compared to all other SNPs with the same minor allele frequency. The p-value represents the fraction of SNPs with a lower Fst value. No individual lung cancer SNP appeared to be significantly differentiated. Fst analysis failed to capture the localized genetic-risk differentiation that has occurred in the Japanese and Druze populations. Combining the p-values revealed no signs that these SNPs have collectively undergone differentiation (p-value = 0.91).
(TIF)

Figure S3 Fst analysis for type 2 diabetes. Global Fst values were calculated for the 16 type 2 diabetes-associated SNPs used in this study. The distribution of p-values did not reveal elevated type 2 diabetes genetic risk differentiation across worldwide populations compared to non-disease associated SNPs. The Fst score for each SNP was compared to all other SNPs with the same minor allele frequency. The combined p-value for these SNPs failed to capture the extreme extent to which genetic-risk differentiation has occurred at a global scale (p-value = 0.30).
(TIF)

Table S1 Type 2 diabetes-associated SNPs replication demographics. The number of times any publication has found each type 2 diabetes SNP used in this study to be associated with the disease is shown. European populations have the most replications across the majority of SNPs associated with type 2 diabetes. Arabic and American Indian populations have the lowest number of replications.
(DOCX)

Table S2 Asian-specific type 2 diabetes effect size. The table compares the overall likelihood ratio for each SNP associated with type 2 diabetes against the Asian-specific likelihood ratio. The Asian-specific likelihood ratio was computed by including only GWASs based in Asian populations. While many of the observed effect sizes did not differ, some were significantly different and may lead to a modified risk estimate for Asian populations.
(DOCX)

Table S3 Genetic risk of type 2 diabetes using Asian GWASs. Most GWASs are based on European cohorts. The effect size of an associated variant may differ across distinct populations. This table compares the combined likelihood ratio for type 2 diabetes (computed using all available GWASs) with the combined likelihood ratio using only GWASs in Asian populations. This approach allowed us to base the effect size of each variant on Asian populations as opposed to mainly European derived populations. Genetic risk for type 2 diabetes is still significantly lower in Asian populations when the effect size for each associated variant is taken from GWASs exclusively based in Asian populations (q-value<0.05).
(DOCX)

Table S4 Replication after removal of outliers. Outliers carry a disproportionate number of risk alleles compared to the rest of their population. For each population, we calculated the interquartile range (IQR) of genetic risk for each individual and removed persons with a genetic risk deviating more than 1.5 IQRs from the population median. The table compares original p-values to the p-value after removal of outliers. A total of 21 out of the original 24 genetic risk differentiation events had p-values<0.006 after removing outliers.
(DOCX)

Table S5 Replication after resampling from known associations. A resampling procedure is used to model the expected genetic risk difference between a population and the rest of the worldwide set of populations combined. However, a GWAS is more likely to detect associations in regions with higher linkage disequilibrium, which is a potential source for bias in detecting genetic risk differentiation. We address this by reproducing the results after randomly drawing only from SNPs previously reported to show association to any phenotype during the resampling step. Genetic risk differentiation is still detected after randomly drawing from other known associations during the resampling step.
(DOCX)

Text S1 Analysis of the genetic basis of disease in the context of worldwide human relationships and migration.
(DOC)
