# Supplementary figures and images for: Analysis of the Genetic Basis of Disease in the Context of Worldwide Human Relationships and Migration
Source: PLoS Genet. 2013 May 23;9(5):e1003447. doi: 10.1371/journal.pgen.1003447 (PMC3662561; doi:10.1371/journal.pgen.1003447)

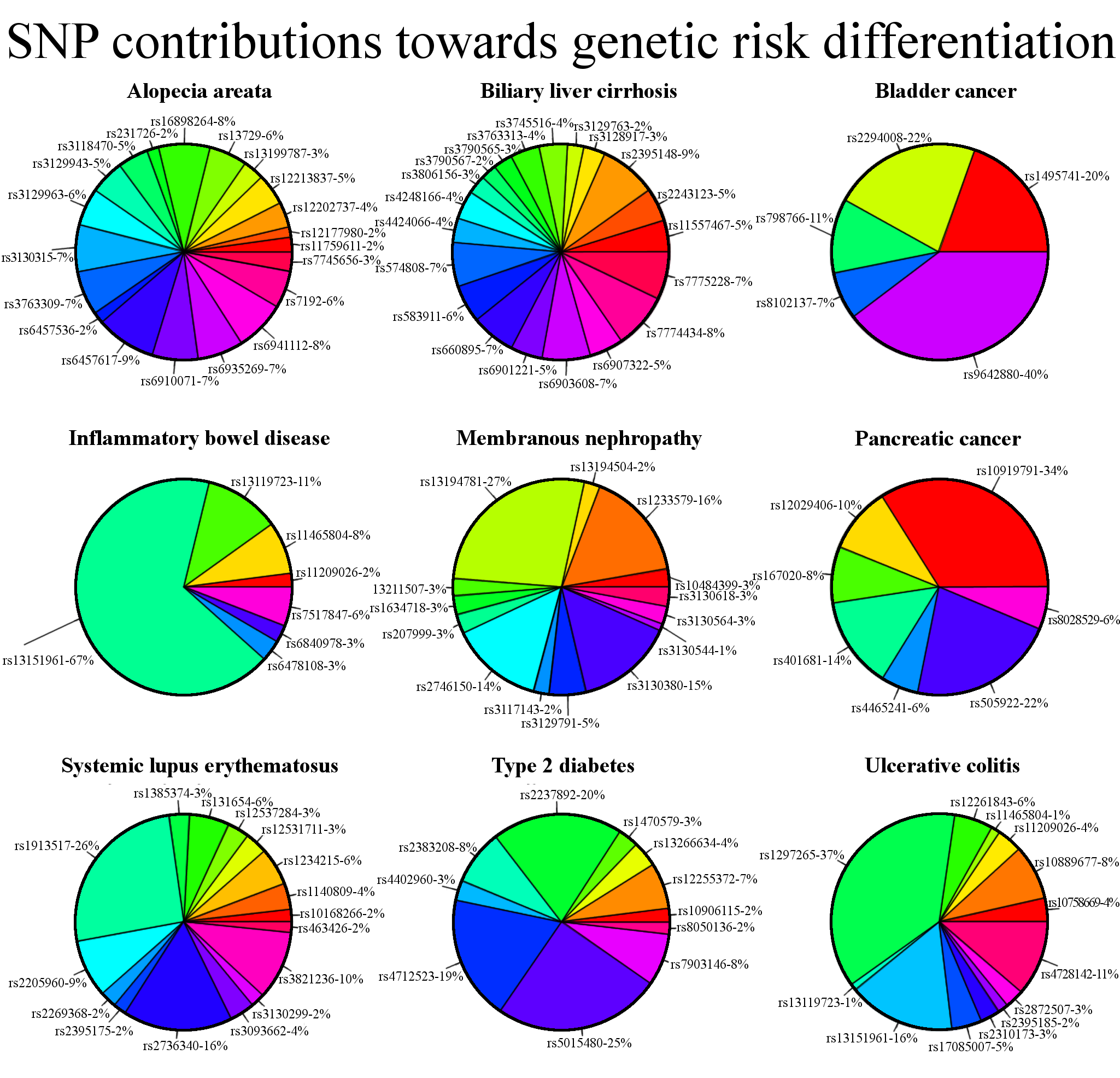

Supplement: Figure S1 — Impact of individual SNPs on genetic risk differentiation. The relative deviation from expected genetic risk attributable to each SNP is shown in each pie chart. The branch in the human phylogeny tree with the most significance for genetic risk differentiation was used to assess the impact of individual SNPs for each disease. The branch selected for alopecia areata includes European, Central South Asian, East Asian, Oceaniac, American, Palestinian, and Druze populations. The branch selected for inflammatory bowel disease includes the Brahui and Makrani populations. The branch selected for pancreatic cancer includes Central South Asian, East Asian, Oceaniac, and American populations. The branch selected for systemic lupus erythematosus includes the Mayan and the Pima populations. The branch selected for type 2 diabetes includes East Asian, Oceaniac, and American populations. The branch selected for ulcerative colitis includes the Brahui and Makrani populations. The branches selected for biliary liver cirrhosis, bladder cancer, and membranous nephropathy are the Druze, Tu, and French Basque populations, respectively. Each branch is compared to all other worldwide populations. Some SNPs have a disproportionate impact on genetic risk. An example is rs13151961, associated with inflammatory bowel disease. (TIF) [file pgen.1003447.s001.tif]

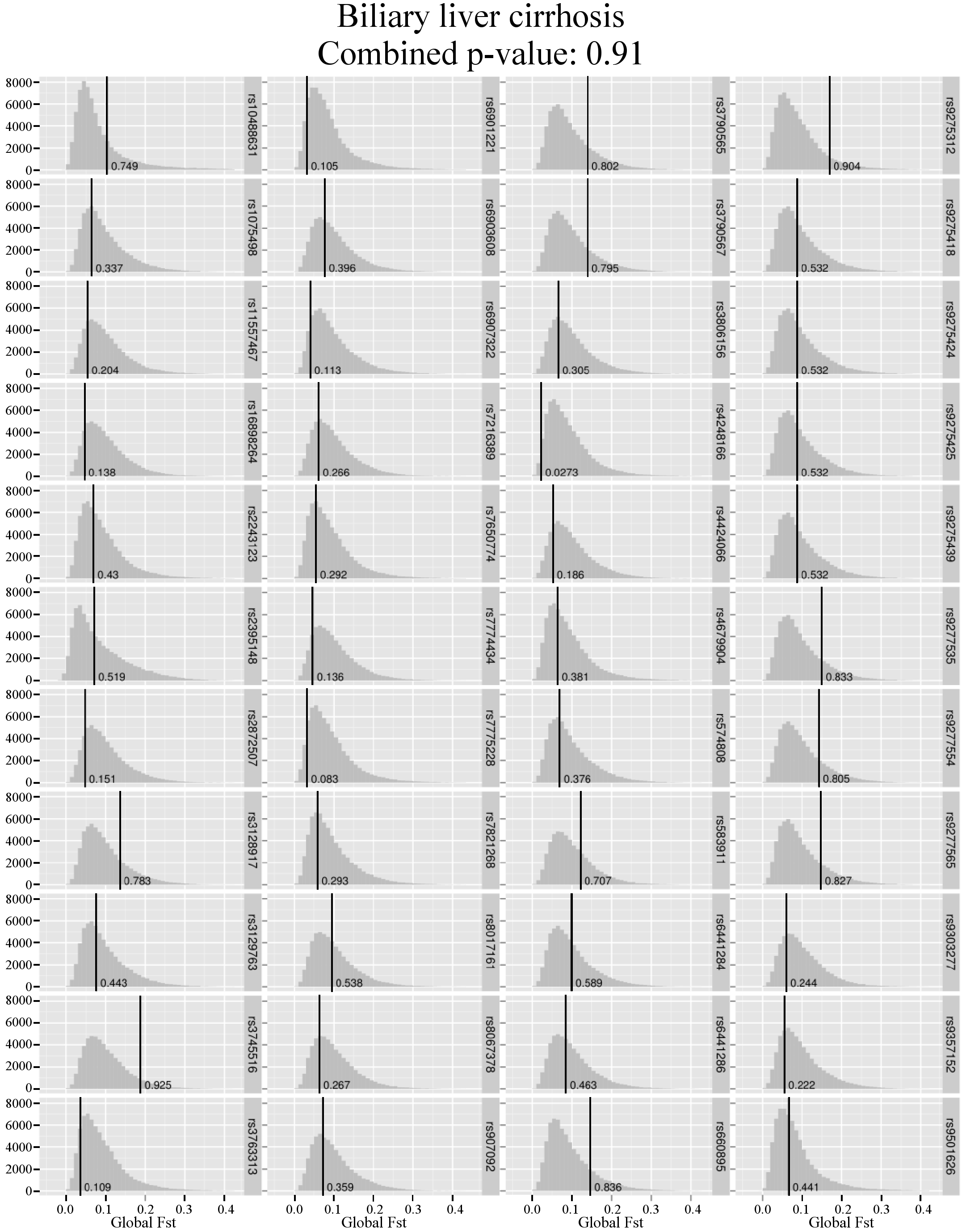

Supplement: Figure S2 — Fst analysis for biliary liver cirrhosis. Global Fst values using the HGDP cohort were calculated for the 44 high confidence biliary liver cirrhosis SNPs used in this study. The Fst score for each SNP was compared to all other SNPs with the same minor allele frequency. The p-value represents the fraction of SNPs with a lower Fst value. No individual lung cancer SNP appeared to be significantly differentiated. Fst analysis failed to capture the localized genetic-risk differentiation that has occurred in the Japanese and Druze populations. Combining the p-values revealed no signs that these SNPs have collectively undergone differentiation (p-value = 0.91). (TIF) [file pgen.1003447.s002.tif]

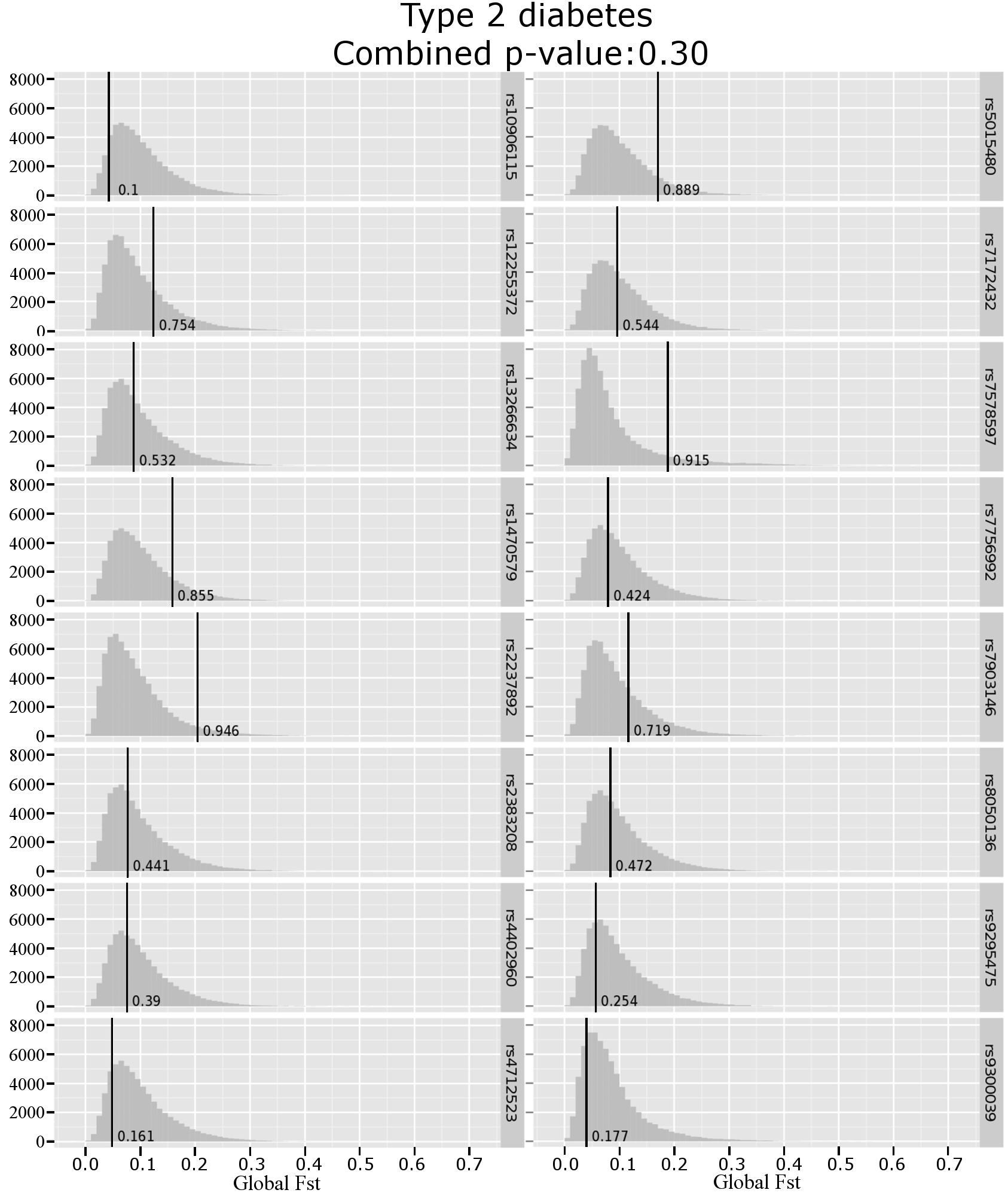

Supplement: Figure S3 — Fst analysis for type 2 diabetes. Global Fst values were calculated for the 16 type 2 diabetes-associated SNPs used in this study. The distribution of p-values did not reveal elevated type 2 diabetes genetic risk differentiation across worldwide populations compared to non-disease associated SNPs. The Fst score for each SNP was compared to all other SNPs with the same minor allele frequency. The combined p-value for these SNPs failed to capture the extreme extent to which genetic-risk differentiation has occurred at a global scale (p-value = 0.30). (TIF) [file pgen.1003447.s003.tif]
