# Supplementary material for: Analysis of the Genetic Basis of Disease in the Context of Worldwide Human Relationships and Migration
Source: PLoS Genet. 2013 May 23;9(5):e1003447. doi: 10.1371/journal.pgen.1003447 (PMC3662561; doi:10.1371/journal.pgen.1003447)
Supplement: Table S1 — Type 2 diabetes-associated SNPs replication demographics. The number of times any publication has found each type 2 diabetes SNP used in this study to be associated with the disease is shown. European populations have the most replications across the majority of SNPs associated with type 2 diabetes. Arabic and American Indian populations have the lowest number of replications. (DOCX) [file pgen.1003447.s004.docx]

| **dbSNP** | **European** | | **Asian** | **African** | **Indian** | **Hispanic** | **Arab** | **American Indian** | **Locus** |
| --- | --- | --- | --- | --- | --- | --- | --- | --- | --- |
| 1470579 | 5 | 5 | | 0 | 0 | 0 | 0 | 0 | IGF2BP2 |
| 2237892 | 2 | 12 | | 0 | 0 | 0 | 0 | 0 | KCNQ1 |
| 2383208 | 0 | 2 | | 0 | 0 | 0 | 0 | 0 | - |
| 4402960 | 19 | 10 | | 2 | 1 | 0 | 0 | 0 | IGF2BP2 |
| 4712523 | 1 | 2 | | 0 | 0 | 0 | 0 | 0 | CDKAL1 |
| 5015480 | 2 | 5 | | 0 | 0 | 0 | 0 | 0 | - |
| 7172432 | 2 | 6 | | 0 | 0 | 0 | 0 | 0 | - |
| 7578597 | 3 | 1 | | 0 | 0 | 0 | 0 | 0 | THADA |
| 7756992 | 6 | 12 | | 0 | 0 | 0 | 0 | 0 | CDKAL1 |
| 7903146 | 75 | 15 | | 7 | 5 | 2 | 2 | 2 | TCF7L2 |
| 8050136 | 12 | 1 | | 1 | 0 | 0 | 0 | 1 | FTO |
| 9295475 | 0 | 2 | | 0 | 0 | 0 | 0 | 0 | CDKAL1 |
| 9300039 | 2 | 0 | | 1 | 0 | 1 | 0 | 0 | - |
| 10906115 | 0 | 1 | | 0 | 0 | 0 | 0 | 0 | - |
| 12255372 | 35 | 8 | | 4 | 5 | 3 | 1 | 1 | TCF7L2 |
| 13266634 | 28 | 14 | | 1 | 0 | 0 | 0 | 0 | SLC30A8 |
