# Supplementary material for: Analysis of the Genetic Basis of Disease in the Context of Worldwide Human Relationships and Migration
Source: PLoS Genet. 2013 May 23;9(5):e1003447. doi: 10.1371/journal.pgen.1003447 (PMC3662561; doi:10.1371/journal.pgen.1003447)
Supplement: Table S2 — Asian-specific type 2 diabetes effect size. The table compares the overall likelihood ratio for each SNP associated with type 2 diabetes against the Asian-specific likelihood ratio. The Asian-specific likelihood ratio was computed by including only GWASs based in Asian populations. While many of the observed effect sizes did not differ, some were significantly different and may lead to a modified risk estimate for Asian populations. (DOCX) [file pgen.1003447.s005.docx]

| **DBSNP** | **Genotype** | **Overall Likelihood Ratio** | **Asian Likelihood Ratio** |
| --- | --- | --- | --- |
| rs1470579 | AA | 0.89 | 0.90 |
|  | AC | 1.07 | 1.06 |
|  | CC | 1.28 | 1.31 |
| rs2237892 | TT | 0.61 | 0.63 |
|  | CT | 0.92 | 0.92 |
|  | CC | 1.21 | 1.21 |
| rs2383208 | GG | 0.71 | 0.71* |
|  | AG | 0.95 | 0.95* |
|  | AA | 1.27 | 1.27* |
| rs4402960 | GG | 0.90 | 0.89 |
|  | TG | 1.06 | 1.08 |
|  | TT | 1.27 | 1.34 |
| rs4712523 | AA | 0.75 | 0.75* |
|  | AG | 1.03 | 1.03* |
|  | GG | 1.43 | 1.43* |
| rs5015480 | TT | 0.89 | 0.89 |
|  | TC | 1.22 | 1.23 |
|  | CC | 1.63 | 1.65 |
| rs7172432 | GG | 0.89 | 0.90 |
|  | AG | 0.98 | 0.98 |
|  | AA | 1.09 | 1.07 |
| rs7578597 | CC | 0.67 | 0.31 |
|  | TC | 0.83 | 0.56 |
|  | TT | 1.02 | 1.01 |
| rs7756992 | AA | 0.83 | 0.79 |
|  | AG | 1.04 | 0.98 |
|  | GG | 1.35 | 1.32 |
| rs7903146 | CC | 0.70 | 0.96 |
|  | TC | 1.17 | 1.16 |
|  | TT | 1.97 | 1.66 |
| rs8050136 | CC | 0.88 | 0.89 |
|  | AC | 1.05 | 0.95 |
|  | AA | 1.26 | 1.00 |
| rs9295475 | AA | 0.89 | 0.89* |
|  | AG | 0.94 | 0.94* |
|  | GG | 1.39 | 1.39* |
| rs9300039 | AA | 0.61 | **NA** |
|  | AC | 0.80 | **NA** |
|  | CC | 1.05 | **NA** |
| rs10906115 | GG | 0.85 | 0.85* |
|  | AG | 0.97 | 0.97* |
|  | AA | 1.10 | 1.10* |
| rs12255372 | GG | 0.81 | 0.97 |
|  | TG | 1.12 | 1.75 |
|  | TT | 1.66 | 1.01 |
| rs13266634 | TT | 0.83 | 0.87 |
|  | TC | 0.95 | 0.98 |
|  | CC | 1.11 | 1.11 |

**NA: The SNP was never verified in an Asian based GWAS and was excluded**

*** no likelihood ratio difference is possible since only Asian-based studies exist**
