# Supplementary material for: Analysis of the Genetic Basis of Disease in the Context of Worldwide Human Relationships and Migration
Source: PLoS Genet. 2013 May 23;9(5):e1003447. doi: 10.1371/journal.pgen.1003447 (PMC3662561; doi:10.1371/journal.pgen.1003447)
Supplement: Table S3 — Genetic risk of type 2 diabetes using Asian GWASs. Most GWASs are based on European cohorts. The effect size of an associated variant may differ across distinct populations. This table compares the combined likelihood ratio for type 2 diabetes (computed using all available GWASs) with the combined likelihood ratio using only GWASs in Asian populations. This approach allowed us to base the effect size of each variant on Asian populations as opposed to mainly European derived populations. Genetic risk for type 2 diabetes is still significantly lower in Asian populations when the effect size for each associated variant is taken from GWASs exclusively based in Asian populations (q-value<0.05). (DOCX) [file pgen.1003447.s006.docx]

|  | **All GWASs** | | | **Asian based GWASs** | | |
| --- | --- | --- | --- | --- | --- | --- |
| **Population** | **Likelihood Ratio** | **P-value** |  | **Likelihood Ratio** | **P-value** |  |
| Bedouin | 1.78 | 1.63 x 10^-2^ |  | 1.46 | 2.84 x 10^-2^ |  |
| Cambodian | 0.53 | 7.91 x 10^-3^ |  | 0.66 | 4.97 x 10^-2^ |  |
| Colombian | 0.46 | 1.13 x 10^-2^ |  | 0.42 | 2.95 x 10^-2^ |  |
| Daur | 0.43 | 1.03 x 10^-3^ |  | 0.47 | 1.91 x 10^-3^ |  |
| Han | 0.52 | 3.39 x 10^-3^ |  | 0.64 | 1.82 x 10^-2^ |  |
| Hezhen | 0.45 | 1.95 x 10^-3^ |  | 0.54 | 1.16 x 10^-2^ |  |
| Japanese | 0.49 | 1.33 x 10^-3^ |  | 0.61 | 1.34 x 10^-2^ |  |
| Lahu | 0.51 | 1.70 x 10^-2^ |  | 0.58 | 3.42 x 10^-2^ |  |
| Mongola | 0.45 | 6.90 x 10^-4^ |  | 0.50 | 2.41 x 10^-3^ |  |
| Mozabite | 2.11 | 4.35 x 10^-3^ |  | 2.27 | 4.91 x 10^-3^ |  |
| A* | 0.89 | 1.15 x 10^-3^ |  | 0.84 | 5.99 x 10^-3^ |  |
| B* | 0.77 | 3.10 x 10^-3^ |  | 0.76 | 3.44 x 10^-3^ |  |
| C* | 0.57 | 1.50 x 10^-4^ |  | 0.61 | 1.56 x 10^-3^ |  |
| D* | 0.55 | 1.90 x 10^-4^ |  | 0.60 | 1.61 x 10^-3^ |  |
| **East Asian** | **0.51** | **2.70 x 10^-4^** |  | **0.60** | **2.71 x 10^-3^** |  |
| E* | 0.47 | 9.29 x 10^-3^ |  | 0.44 | 2.35 x 10^-2^ |  |
| Naxi | 0.46 | 3.53 x 10^-3^ |  | 0.60 | 3.47 x 10^-2^ |  |
| Oroqen | 0.45 | 2.41 x 10^-3^ |  | 0.53 | 1.00 x 10^-2^ |  |
| Palestinian | 1.82 | 6.75 x 10^-3^ |  | 1.62 | 4.96 x 10^-3^ |  |
| She | 0.43 | 2.65 x 10^-3^ |  | 0.48 | 5.24 x 10^-3^ |  |
| Surui | 0.42 | 2.23 x 10^-2^ |  | 0.39 | 4.63 x 10^-2^ |  |
| Tu | 0.51 | 4.51 x 10^-3^ |  | 0.61 | 2.17 x 10^-2^ |  |
| Tujia | 0.49 | 6.11 x 10^-3^ |  | 0.58 | 2.27 x 10^-2^ |  |
| Xibo | 0.51 | 4.97 x 10^-3^ |  | 0.67 | 4.83 x 10^-2^ |  |
| Yakut | 0.63 | 1.38 x 10^-2^ |  | 0.60 | 5.46 x 10^-2^ |  |
| Yizu | 0.43 | 1.47 x 10^-3^ |  | 0.54 | 9.89 x 10^-3^ |  |

**A* Europe, Central South Asia, East Asia, Oceania, and America**

**B* Central South Asia, East Asia, Oceania, and America**

**C* East Asia, Oceania, and America**

**D* East Asia and America**

**H* Colombia, Karitiana, and Surui**
