# Supplementary material for: Analysis of the Genetic Basis of Disease in the Context of Worldwide Human Relationships and Migration
Source: PLoS Genet. 2013 May 23;9(5):e1003447. doi: 10.1371/journal.pgen.1003447 (PMC3662561; doi:10.1371/journal.pgen.1003447)
Supplement: Table S4 — Replication after removal of outliers. Outliers carry a disproportionate number of risk alleles compared to the rest of their population. For each population, we calculated the interquartile range (IQR) of genetic risk for each individual and removed persons with a genetic risk deviating more than 1.5 IQRs from the population median. The table compares original p-values to the p-value after removal of outliers. A total of 21 out of the original 24 genetic risk differentiation events had p-values<0.006 after removing outliers. (DOCX) [file pgen.1003447.s007.docx]

| **Disease** | **Population** | **Original**  **P-Value** | **P-value:**  **No Outliers** | **Outliers Removed** |
| --- | --- | --- | --- | --- |
| Alopecia areata | Mozabite | 1.00 x 10^-5^ | 1.00 x 10^-5^ | 52 |
| Alopecia areata | G* | 5.70 x 10^-4^ | 5.00 x 10^-4^ | 52 |
| Biliary liver cirrhosis | Druze | 5.70 x 10^-4^ | 5.69 x 10^-3^ | 45 |
| Biliary liver cirrhosis | Japanese | 3.70 x 10^-4^ | 7.45 x 10^-3^ | 45 |
| Bladder cancer | Tu | 4.90 x 10^-4^ | 4.70 x 10^-3^ | 54 |
| Inflammatory bowel disease | Balochi | 1.00 x 10^-5^ | 1.00 x 10^-5^ | 91 |
| Inflammatory bowel disease | Burusho | 4.30 x 10^-4^ | 6.05 x 10^-1^ | 91 |
| Inflammatory bowel disease | Makrani | 1.50 x 10^-4^ | 3.00 x 10^-5^ | 91 |
| Inflammatory bowel disease | E* | 1.50 x 10^-4^ | 1.00 x 10^-5^ | 91 |
| Inflammatory bowel disease | Palestinian | 5.00 x 10^-5^ | 4.74 x 10^-1^ | 91 |
| Inflammatory bowel disease | Sindhi | 5.00 x 10^-5^ | 4.25 x 10^-1^ | 91 |
| Membranous nephropathy | French Basque | 1.00 x 10^-5^ | 2.90 x 10^-3^ | 67 |
| Pancreatic cancer | A* | 1.00 x 10^-5^ | 1.00 x 10^-5^ | 34 |
| Pancreatic cancer | B* | 1.00 x 10^-5^ | 1.00 x 10^-5^ | 34 |
| Pancreatic cancer | F* | 3.50 x 10^-4^ | 3.30 x 10^-4^ | 34 |
| Pancreatic cancer | Yoruba | 3.10 x 10^-4^ | 3.90 x 10^-4^ | 34 |
| Systemic lupus erythematosus | Maya + Pima* | 3.30 x 10^-4^ | 2.50 x 10^-4^ | 45 |
| Type 2 diabetes | B* | 3.10 x 10^-4^ | 1.10 x 10^-4^ | 48 |
| Type 2 diabetes | C* | 1.50 x 10^-4^ | 1.90 x 10^-4^ | 48 |
| Type 2 diabetes | D* | 1.90 x 10^-4^ | 3.10 x 10^-4^ | 48 |
| Type 2 diabetes | East Asia* | 2.70 x 10^-4^ | 1.49 x 10^-3^ | 48 |
| Ulcerative colitis | Balochi | 1.00 x 10^-5^ | 1.00 x 10^-5^ | 55 |
| Ulcerative colitis | E* | 1.00 x 10^-5^ | 3.00 x 10^-5^ | 55 |
| Ulcerative colitis | Sindhi | 1.00 x 10^-5^ | 3.00 x 10^-5^ | 55 |

**A* Europe, Central South Asia, East Asia, Oceania, and America**

**B* Central South Asia, East Asia, Oceania, and America**

**C* East Asia, Oceania, and America**

**D* East Asia and America**

**E* Brahui, Makrani**

**F* Yoruba, Mandenka**

**G* Europe, Central South Asia, East Asia, Oceania, and America, Palestinian, Druze**
