# Supplementary material for: Analysis of the Genetic Basis of Disease in the Context of Worldwide Human Relationships and Migration
Source: PLoS Genet. 2013 May 23;9(5):e1003447. doi: 10.1371/journal.pgen.1003447 (PMC3662561; doi:10.1371/journal.pgen.1003447)
Supplement: Table S5 — Replication after resampling from known associations. A resampling procedure is used to model the expected genetic risk difference between a population and the rest of the worldwide set of populations combined. However, a GWAS is more likely to detect associations in regions with higher linkage disequilibrium, which is a potential source for bias in detecting genetic risk differentiation. We address this by reproducing the results after randomly drawing only from SNPs previously reported to show association to any phenotype during the resampling step. Genetic risk differentiation is still detected after randomly drawing from other known associations during the resampling step. (DOCX) [file pgen.1003447.s008.docx]

| **Disease** | **Population** | **Original**  **P-Value** | **Known Associations**  **P-value** | **SNPs** |
| --- | --- | --- | --- | --- |
| Alopecia areata | Mozabite | 1.00 x 10^-5^ | 1.00 x 10^-5^ | 26 |
| Alopecia areata | G* | 5.70 x 10^-4^ | 7.50 x 10^-4^ | 38 |
| Biliary liver cirrhosis | Druze | 5.70 x 10^-4^ | 1.69 x 10^-3^ | 30 |
| Biliary liver cirrhosis | Japanese | 3.70 x 10^-4^ | 2.77 x 10^-3^ | 27 |
| Bladder cancer | Tu | 4.90 x 10^-4^ | 5.30 x 10^-4^ | 7 |
| Inflammatory bowel disease | Balochi | 1.00 x 10^-5^ | 5.00 x 10^-5^ | 11 |
| Inflammatory bowel disease | Burusho | 4.30 x 10^-4^ | 2.49 x 10^-3^ | 9 |
| Inflammatory bowel disease | Makrani | 1.50 x 10^-4^ | 1.29 x 10^-3^ | 10 |
| Inflammatory bowel disease | E* | 1.50 x 10^-4^ | 3.00 x 10^-5^ | 11 |
| Inflammatory bowel disease | Palestinian | 5.00 x 10^-5^ | 3.90 x 10^-4^ | 11 |
| Inflammatory bowel disease | Sindhi | 5.00 x 10^-5^ | 3.10 x 10^-4^ | 10 |
| Membranous nephropathy | French Basque | 1.00 x 10^-5^ | 7.10 x 10^-4^ | 17 |
| Pancreatic cancer | A* | 1.00 x 10^-5^ | 1.00 x 10^-5^ | 8 |
| Pancreatic cancer | B* | 1.00 x 10^-5^ | 1.00 x 10^-5^ | 8 |
| Pancreatic cancer | F* | 3.50 x 10^-4^ | 2.30 x 10^-4^ | 9 |
| Pancreatic cancer | Yoruba | 3.10 x 10^-4^ | 1.50 x 10^-4^ | 9 |
| Systemic lupus erythematosus | Maya + Pima* | 3.30 x 10^-4^ | 3.50 x 10^-4^ | 29 |
| Type 2 diabetes | B* | 3.10 x 10^-4^ | 2.50 x 10^-4^ | 14 |
| Type 2 diabetes | C* | 1.50 x 10^-4^ | 1.30 x 10^-4^ | 14 |
| Type 2 diabetes | D* | 1.90 x 10^-4^ | 1.30 x 10^-4^ | 14 |
| Type 2 diabetes | East Asia* | 2.70 x 10^-4^ | 7.00 x 10^-5^ | 14 |
| Ulcerative colitis | Balochi | 1.00 x 10^-5^ | 1.00 x 10^-5^ | 27 |
| Ulcerative colitis | E* | 1.00 x 10^-5^ | 1.00 x 10^-5^ | 27 |
| Ulcerative colitis | Sindhi | 1.00 x 10^-5^ | 3.00 x 10^-5^ | 27 |

**A* Europe, Central South Asia, East Asia, Oceania, and America**

**B* Central South Asia, East Asia, Oceania, and America**

**C* East Asia, Oceania, and America**

**D* East Asia and America**

**E* Brahui, Makrani**

**F* Yoruba, Mandenka**

**G* Europe, Central South Asia, East Asia, Oceania, and America, Palestinian, Druze**
