# Supplementary material for: Analysis of the Genetic Basis of Disease in the Context of Worldwide Human Relationships and Migration
Source: PLoS Genet. 2013 May 23;9(5):e1003447. doi: 10.1371/journal.pgen.1003447 (PMC3662561; doi:10.1371/journal.pgen.1003447)
Supplement: Text S1 — Analysis of the genetic basis of disease in the context of worldwide human relationships and migration. (DOC) [file pgen.1003447.s009.doc]

Supporting online material: Analysis of the Genetic Basis of Disease in the Context of Worldwide Human Relationships and Migration

Comparison with Fst

Although Fst is a traditional approach for detecting population differentiation , its use for detecting genetic risk differentiation is inappropriate for two reasons. First, it does not account for the effect size of each susceptibility allele. Fst treats all variants equally instead of weighing variants proportionately on how much they increase/decreases genetic risk of disease. In addition, there is a well-known correlational structure within the populations studied in HGDP . Our Genetic Risk Variation (GRV) method found significant genetic-risk differentiation for biliary liver cancer (BLC) in 2 populations (Error: Reference source not foundB), yet Fst analysis failed to detect significant deviations from the background (Figure S2). This finding is not surprising in BLC, as global Fst values are not likely to detect the localized risk differentiation we observed. However, Figure S3 shows that Fst analysis also failed to detect genetic risk differentiation in type 2 diabetes despite high levels of worldwide differentiation found by our method (Error: Reference source not foundA).

GRV accounts for the effect size of each genotype on disease and controls for genetic drift. It estimates the geographic location where disease risk is most likely to have changed compared to other populations, as well as its position in the phylogenetic tree. It relies on q-value computations (the expected proportion of false positives incurred when labeling a particular p-value significant) to control for multiple hypotheses.

Coop et al. also developed a method to identify allele frequency correlations with environmental features . However, their method correlates allele frequency to environment, while genetic risk estimates are a function of genotype frequencies. The goal of our study was to detect genetic risk differentiation across worldwide populations. We have emphasized cases where risk differentiation exceeds what would be expected under genetic drift.

Assessing the Impact of Individual SNPs

Figure S1 shows the relative impact of each SNP on overall levels of genetic risk differentiation in nine diseases. We chose one branch in the human phylogeny tree per disease in order to assess each SNP’s impact. Each branch was compared to all other worldwide populations. The branch selected to assess the impact of each individual SNP was chosen as the branch with the strongest significance (lowest q-value) with respect to each disease. The expected risk difference between two populations was ascertained by the method used for multiple SNPs, but using one SNP at a time. As before, the selected branch for a disease is compared to all other worldwide populations by measuring deviation of observed genetic risk difference (between the two populations) with the expected genetic risk difference. SNPs contributing to decreased significance for risk differentiation were excluded. Each deviation measurement was normalized by dividing by the sum of all deviation measurements and multiplying by 100 to produce percent values. Figure S1 represents the percent of deviation from the expected genetic risk that is attributable to each SNP.

Phenotypes Transcend Population Boundaries

Populations are typically examined independently without combining closely related groups of people. In reality, phenotypes are seldom strictly divided across populations. It is evident that the degree of phenotype similarity observed across different populations varies. Some characteristic phenotypes may be observed in almost all populations on a continent, such as periocular anatomy in East Asia. Traits such as disease susceptibility are not as easily observed and it is difficult to know in advance how to group different populations when searching for genetic risk deviations. This is why iteratively grouping the two most phenotypically closely related populations to search for genetic risk differentiation not only increases sample size, but represents abandoning the common practice of studying traits within established population boundaries. This can also lead to revelations regarding points on the human phylogenetic tree at which genetic risk differentiation occurred. For example, studies have indicated that East Asians have a lower genetic risk for type 2 diabetes . We found that American populations also have a lower genetic risk for this disease. However, the ancestral population common to East Asians and Americans is not the most likely to have actually undergone genetic risk differentiation (Error: Reference source not foundA). One of the insights gained from using a phylogenetic tree in this analysis is that it is more likely that an ancestor of East Asians and a different ancestor of Native American populations independently experienced a genetic risk differentiation event in a trajectory toward lower risk for type 2 diabetes. This possibility is counter to the idea that Native American and East Asian populations merely inherited lower genetic risk prior to the branching of the two populations.

Limitations

Prevalence information was not included in this study. Genetic risk does not necessarily correlate with disease prevalence in practice or theory. Intuitively, one might expect prevalence to be higher in populations with higher predicted genetic risk, but we hypothesize that environmental risk and genetic risk can just as easily be negatively correlated. Higher genetic risk may accumulate in an environment strongly disfavoring disease as there is no selection pressure to act against the spread of increased genetic risk for disease. Likewise, genetic resistance to disease may develop as a natural result of living in an environment strongly favoring its occurrence. In addition, if the environment has recently undergone change or a population is physically displaced, risk/prevalence ratios would change. Such an analysis is more appropriate for a study exploring gene-environment interactions on disease in the context of differences in genetic risk. It has been shown that the environment has interacted with many disease-associated loci in different ways across distinct human populations .

This analysis does not incorporate information about the environment, which may further explain relationships between disease incidence and genetic risk. There is already work underway to comprehensively associate environmental factors with disease . Repeating such work in the context of genetic risk estimates would likely lead to an increased ability to predict overall risk of disease as it would include a variety of environmental factors as well as all known disease associated SNPs.

A large fraction of mutations differentiated across populations are compensatory in long-term evolution . The role of compensatory mutations in recent human evolution is unlikely to be as significant as in long-term evolution due to the relatively small effect size of common disease-associated SNPs. Yet the role of compensatory mutations in recent human evolution is not well established. In our study, genetic risk was measured with alleles increasing and decreasing genetic risk collectively. If variants increasing genetic risk in a particular population are present, they will not be detected if their effect is mitigated by other variants decreasing risk, potentially limiting our findings.

Because the majority of disease-associated SNPs used in this study were tag SNPs, it is also possible that varied LD patterns across populations would stop the tag SNP from tagging the correct disease-associated SNP in a different population. The best way to combat the possibility of false positives due to this problem is to increase the minimum number of SNPs required to represent a disease. In addition, the majority of disease-associated SNPs contain other reported associations in LD. We attempted to pick the associated SNP closest to the true causative SNP, thereby further decreasing the likelihood of an LD gap across distinct populations. We accomplished this task by examining effect size information for each of the associated SNPs in LD and picking the SNP with the largest effect size from the LD block while discarding the others. We considered only SNPs with p-values of association below 10^-6^. In addition, the SNPs in this study were replicated across two or more populations (see Table S1 for type 2 diabetes), thereby making it more likely that they were in strong linkage disequilibrium to the causal SNPs and rendering them appropriate proxies.

A more obvious problem is the missing heritability problem. Known variants explain very little of the observed risk for complex disease. While there are many potential explanations for this problem (copy-number polymorphisms, multiple loci with very small individual effects, pervasive epistatic effects, etc.), it will take considerable further research to explore them all . Further research will likely identify rare variants associated with many diseases. The variants in this study have a specific allele frequency and effect size combination that makes them detectable in the populations in which the GWASs were conducted. Our results reflect this particular category of disease-associated SNPs.

Despite the low predictive power caused by the missing heritability problem, genetic risk differentiation was detected across multiple diseases. In addition, the fact that most GWASs are conducted in European populations makes it difficult to properly assess risk in non-Europeans. For example, if increased risk is detected in Europeans, it is possible that Africans have undiscovered variants that offset the observed difference in risk reported in this study. However, it would still be important to note that common variants increasing the genetic risk of disease primarily in European populations show strong signs of genetic risk differentiation on a global scale, as is the case for type 2 diabetes.

**1. Reynolds J, Weir BS, Cockerham CC (1983) Estimation of the coancestry coefficient: basis for a short-term genetic distance. Genetics 105: 767-779.**

**2. Li JZ, Absher DM, Tang H, Southwick AM, Casto AM, et al. (2008) Worldwide human relationships inferred from genome-wide patterns of variation. Science 319: 1100-1104.**

**3. Storey JD, Tibshirani R (2003) Statistical significance for genomewide studies. Proc Natl Acad Sci U S A 100: 9440-9445.**

**4. Coop G, Witonsky D, Di Rienzo A, Pritchard JK Using environmental correlations to identify loci underlying local adaptation. Genetics 185: 1411-1423.**

**5. Klimentidis YC, Abrams M, Wang J, Fernandez JR, Allison DB Natural selection at genomic regions associated with obesity and type-2 diabetes: East Asians and sub-Saharan Africans exhibit high levels of differentiation at type-2 diabetes regions. Hum Genet 129: 407-418.**

**6. Kathiresan S, Melander O, Anevski D, Guiducci C, Burtt NP, et al. (2008) Polymorphisms associated with cholesterol and risk of cardiovascular events. N Engl J Med 358: 1240-1249.**

**7. Wacholder S, Hartge P, Prentice R, Garcia-Closas M, Feigelson HS, et al. Performance of common genetic variants in breast-cancer risk models. N Engl J Med 362: 986-993.**

**8. Pedersen NL Reaching the limits of genome-wide significance in Alzheimer disease: back to the environment. JAMA 303: 1864-1865.**

**9. Patel CJ, Bhattacharya J, Butte AJ An Environment-Wide Association Study (EWAS) on type 2 diabetes mellitus. PLoS One 5: e10746.**

**10. Camps M, Herman A, Loh E, Loeb LA (2007) Genetic constraints on protein evolution. Crit Rev Biochem Mol Biol 42: 313-326.**

**11. Eichler EE, Flint J, Gibson G, Kong A, Leal SM, et al. Missing heritability and strategies for finding the underlying causes of complex disease. Nat Rev Genet 11: 446-450.**
